# PARKINSON’S DISEASE AND STIGMA: A MULTIPLE CASE STUDY

**DOI:** 10.1093/geroni/igac059.2900

**Published:** 2022-12-20

**Authors:** Beth Mastel-Smith, Melinda Hermanns

**Affiliations:** The University of Texas at Tyler, Tyler, Texas, United States; The University of Texas at Tyler, Tyler, Texas, United States

## Abstract

Parkinson’s disease (PD) is a neurodegenerative disorder caused by dopamine deficiency. PD affects emotional and physical health and causes people with PD and caregivers to experience stigma. Four types of stigma have been described: public, associated, self-, and structural. The aims of this study were to explore (a) how stigma is experienced by people with PD and caregivers, (b) whether stigma towards people with PD and caregivers is represented in the literature and social media and (c) examine whether findings support Stigma Theory and theoretical propositions. This was a multi-case qualitative study. Cases included a literature review, interviews with people with PD and caregivers, and examination of social media posts. A purposive sample included seven people with PD and five caregivers who were interviewed online using an interview guide. The study was approved by the University IRB and participants signed consent. Thematic analysis identified codes which aligned with conceptual definitions of Stigma Theory. Findings revealed that public stigma was presented in all four cases. Self- and structural stigma were represented in the literature and interviews with people with PD and caregivers. Associated stigma appeared in the literature and from caregivers’ descriptions. Three of six theoretical propositions were supported. Four types of stigma toward people with PD were prevalent and interrelationships between types of stigma noted. A multi-pronged approach to reducing stigma is needed at the individual, organizational and community levels. Research regarding stigma experienced by diverse populations and the health effects of stigma for people with PD is recommended.

